# Resident duty hour modification affects perceptions in medical education, general wellness, and ability to provide patient care

**DOI:** 10.1186/s12909-016-0703-4

**Published:** 2016-07-13

**Authors:** Andrew Moeller, Jordan Webber, Ian Epstein

**Affiliations:** Division of Cardiology, Department of Medicine, Dalhousie University & Capital Health, QEII – Halifax Infirmary Site, 1796 Summer Street, B3H 3A6 Halifax, NS Canada; Department Medicine, Dalhousie University & Capital Health, QEII Health Sciences Centre, VG Site, 1276 South Park St, Halifax, NS B3H 2Y9 Canada

**Keywords:** Resident duty hour modification, Post-graduate, Residency, Curriculum, Quantitative research

## Abstract

**Background:**

Resident duty hours have recently been under criticism, with concerns for resident and patient well-being. Historically, call shifts have been long, and some residency training programs have now restricted shift lengths. Data and opinions about the effects of such restrictions are conflicting. The Internal Medicine Residency Program at Dalhousie University recently moved from a traditional call structure to a day float/night float system. This study evaluated how this change in duty hours affected resident perceptions in several key domains.

**Methods:**

Senior residents from an internal medicine training program in Canada responded to an anonymous online survey immediately before and 6 months after the implementation of duty hour reform. The survey contained questions relating to three major domains: resident wellness, ability to deliver quality health care, and medical education experience. Mean pre- and post-intervention scores were compared using the *t*-test for paired samples.

**Results:**

Twenty-three of 27 (85 %) senior residents completed both pre- and post-reform surveys. Residents perceived significant changes in many domains with duty hour reform. These included improved general wellness, less exposure to personal harm, fewer feelings of isolation, less potential for error, improvement in clinical skills expertise, increased work efficiency, more successful teaching, increased proficiency in medical skills, more successful learning, and fewer rotation disruptions.

**Conclusions:**

Senior residents in a Canadian internal medicine training program perceived significant benefits in medical education experience, ability to deliver healthcare, and resident wellness after implementation of duty hour reform.

**Electronic supplementary material:**

The online version of this article (doi:10.1186/s12909-016-0703-4) contains supplementary material, which is available to authorized users.

## Background

Resident duty hours have received national and international attention over the last several years. Residents provide important hands-on 24/7 patient care coverage in hospitals with individual shift lengths frequently in excess of 24 h without restful sleep, while having a dual role of both care provider and learner.

Sleep deprivation and the number of hours worked by a resident physician have been linked to significant medical errors and attention dysfunction [1], motor vehicle collisions [2–4], adverse events [5], and impaired working memory capacity [6]. Studies have shown a correlation between long work hours and increased workplace injury [7] and burnout [8]. Sleep deprivation has been linked to decreased technical proficiency [9–11], impaired neuropsychological testing and less accurate ECG interpretation [12].

In order to address several of the issues outlined above, there have been national and international efforts to reform resident duty hours. Resident duty hours have been restricted in many international jurisdictions including Europe (40–52.5 h per week) and the United States (80 h per week), and shift length has been restricted across Canada from a previous maximum of 32-hour shifts to 26 h. In Quebec, duty hour length has been shortened to a maximum of 16 h [13]. There is conflicting evidence as to whether such reforms have improved safety or patient outcomes. Parshuram et al. (2015) showed that shorter ICU shifts resulted in increased medical errors and worsened resident satisfaction [14]. Conversely, in 2004 Landrigan et al. studied the direct effect of duty hour length on objective measures such as serious medical errors, and found frequent shifts in excess of 24 h were associated with significantly more serious medical errors compared to shorter shifts [15]. A recent large, rigourous study, conducted with the assistance of the Accreditation Council for Graduate Medical Education, found that there were no significant differences in mortality and readmissions before and after the implementation of the 2011 ACGME duty hour reforms [16].

In 2013, a National Steering Committee of Resident Duty Hours was formed by the Royal College of Physicians and Surgeons of Canada, which was funded by Health Canada, to form a unified consensus regarding resident duty hours [13]. Their recommendation was that “duty periods of twenty four or more consecutive hours without restorative sleep should be avoided,” and “pilot projects should be developed, supported, and catalogued to consider a range of educational tools and innovative scheduling systems [13].”

As a result of the recommendation, the Residency Programming Committee (RPC) for the Internal Medicine training program at Dalhousie University in Halifax, Canada approved a trial of resident duty hour reform for senior residents on the emergency department consultation service. The trial involved changing the Senior Medicine Resident call shift from the previous 24-hour coverage to a day-float and night-float schedule. The details of this call structure are described in the methods section. Due to the conflicting data existing on the topic of resident duty hours, we administered a previously validated, evidence-based questionnaire prior to and after initiation of duty hour reform to assess senior residents’ perceptions of the implications of duty hour reform [17].

## Methods

The Dalhousie Internal Medicine call schedule structure is designed to respond to site-specific requirements. There is a dedicated emergency department consultation team with 24-hour service. Weekday coverage is scheduled in a day-float and night-float system, with weekend coverage in traditional 24-hour shifts. Senior resident responsibilities include reviewing all consultations in the emergency department, providing support for more than 50 admitted inpatients on the medical teaching unit, 8–9 patients in an intermediate care unit, and leading the code blue team for the hospital. In January 2014, the weekend Senior Medicine Resident call shift was changed from the previous 24-hour coverage to a day-float and night-float schedule, similar to the weekday format, with an 11-hour day shift and 15-hour night shift. There was a scheduled, dedicated 1-hour overlap structured at shift change for effective handover. This change was recommended by the Residency Programming Committee, with resident surveys to be completed for quality assurance. The study design was informally presented to Dalhousie University Research Ethics board members. The Tri-Council Policy Statement (TCPS2) governing research ethics in Canada suggests that while research must undergo ethical review, program evaluation and qualitative improvements studies do not fall under the auspices of the TCPS or institutional Research Ethics Boards (TCPS2, Article 2.5). This project was described as quality improvement and therefore a formal research ethics board approval was not recommended.

### Study design

This is a prospective cohort study similar to that conducted by Fabreau et al. in 2013 [17]. The sample participants are all Senior Internal Medicine Residents defined as being in their second or third year of training at Dalhousie University. The primary author emailed all of the Senior Medicine Residents on behalf of the research team. Informed consent was obtained and all Senior Residents consented and were initially enrolled in the study. Residents completed anonymous web-based surveys prior to the change in January 2014 and then in June of 2014, after 5 months of experience with the new schedule. Participants received email communication on January 7th and January 14th, 2014 as notification to complete the preliminary survey. The same participants received email communication on June 21st and June 28th as notification to complete the final post-intervention survey. The validated survey was adapted from that designed by Fabreau et al. with expressed consent [17]. A total of 47 questions were answered on a 5-point Likert scale, measuring the extent to which respondents agreed or disagreed with statements relating to three major domains: senior residents’ wellness (15 items), ability to deliver quality health care (14 items), and medical education experience (18 items) (Additional File 1). Details of these categories are highlighted in Table 1. Senior resident wellness includes indicators of general wellbeing, exposure to harm, relationships, and emotional experience during training, as affected by duty hours. Health care delivery as a category, focuses on residents’ perceived effect prolonged call shifts have upon practical clinical skills, continuity of patient care, potential for clinical error, and overall work efficiency. Medical education experience identifies various facets of the residents’ perceived learning environment and structure during training. This includes their learned ability to teach and be taught through formal and informal educational sessions, learning of technical medical skills with adequate attending physician supervision, all while minimizing rotation disruption. Three questions from the original survey were omitted and two were added due to differences in the senior resident responsibilities between the University of Calgary and Dalhousie University. Each of the three major domains contained five sub-domains (Table 1). Demographic information was not collected from participants. However, we controlled for year of experience by isolating the sample population to residents in their second and third year of training. The study population was relatively small and the inherent variability of the demographics was outside the control of the study investigators. Therefore, these factors were excluded from our primary data acquisition, as it would not be utilized in our primary outcome analyses.

**Table 1:** The three main subdomains are broken down into 5 subdomains listed below

Domains	Sub-domains
Senior Resident Wellness	• Allows general wellness
	• Allows exposure to harm
	• Causes conflicting role demands
	• Allows healthy relationships
	• Causes feelings of isolation
Ability to Deliver Health Care	• Allows potential for error
	• Allows clinical skills expertise
	• Allows continuity of patient care
	• Causes expenditure of emotional labour
	• Allows work efficiency
Medical Education Experience	• Allows successful teaching
	• Allows medical skills proficiency
	• Allows successful learning
	• Allows staff physician supervision
	• Causes rotation disruptions

The primary outcome was the change in Senior Medicine Residents’ perceptions of resident wellness, ability to deliver quality health care and medical education experience both pre- and post-intervention.

### Data collection

Surveys were distributed by email from the study investigators. Consent was implied by responding to the survey. Reminder emails were sent to optimize the rate of reply. Surveys were administered using the computer program *Opinio*™. Data were collected and stored in an electronic form through a secure database in the Department of Medicine. A separate Department of Medicine employee, not involved in the survey, anonymized the data points with unique identifiers. Only study investigators and an associated statistician had access to data.

### Statistical analysis

The Dalhousie University Research Methods Unit (RMU) performed the statistical analysis for the quantitative data. Investigators had access to data only after statistical analysis was complete. Individual Likert items were grouped into 15 scales, 5 for each domain. Scale scores were calculated by summing the individual Likert item scores and dividing by the number of items for a standardized scale score between 1 and 5. Only respondents who completed both the pre and post-surveys were included in the analysis. Scale scores are presented as means and compared using the *t*-test for paired samples, and a p-value of <0.05 was used to indicate statistically significant difference between pre- and post-scores.

## Results

All of the 27 senior residents responded to the pre-intervention survey. Twenty-three of the 27 (85 %) responded to the post-intervention survey. Therefore 23 senior residents formed the final, paired cohort from which data were analyzed (Table 2).

**Table 2:** Survey results comparing pre- and post-duty hour modification survey results. Mean values represent location on a traditional 5-point Likert scale. Statistically significant differences are identified in bold text

Aspects of senior resident self/work affected by 24 h call shifts	Pre-intervention	Post-intervention		Number perceiving improvement
Senior resident wellness	Mean (SD)	Mean (SD)	p-value	# (%)
Allows general wellness	2.70 (0.64)	3.06 (0.71)	**0.04**	**13 (56.52)**
Allows exposure to personal harm	3.72 (0.82)	2.91 (1.06)	**0.0003**	**17 (73.91)**
Causes conflicting role demands	3.27 (0.83)	2.85 (0.68)	0.08	13 (56.52)
Allows healthy relationships	2.70 (0.88)	3.13 (0.81)	0.09	10 (43.48)
Causes feelings of isolation	3.39 (1.03)	2.96 (1.07)	**0.02**	**9 (39.13)**
Ability to deliver health care				
Allows potential for error	3.15 (0.76)	2.47 (0.68)	**0.0001**	**17 (73.91)**
Allows clinical skill expertise	3.39 (0.72)	3.91 (0.52)	**0.0004**	**17 (73.91)**
Allows continuity of patient care	4.13 (0.87)	4.22 (0.6)	0.6	6 (26.09)
Causes expenditure of emotional labour	1.84 (0.74)	1.75 (0.56)	0.58	8 (34.78)
Allows work efficiency	3.72 (0.75)	4.14 (0.33)	**0.001**	**14 (60.87)**
Medical education experience				
Allows successful teaching	3.12 (0.82)	3.81 (0.59)	**0.0009**	**17 (73.91)**
Allows medical skills proficiency	3.58 (0.88)	4.04 (0.73)	**0.001**	**16 (69.57)**
Allows successful learning	3.52 (0.57)	4.00 (0.56)	**0.003**	**15 (65.22)**
Allows staff physician supervision	3.23 (0.75)	3.38 (0.59)	0.37	7 (30.43)
Causes rotation disruptions	3.29 (0.88)	2.84 (0.85)	**0.04**	**14 (60.87)**

Senior residents found that the change to resident duty hours improved their perception of medical education experiences. In total, 4 out of 5 of the sub-domains in medical education experience showed a statistically significant positive difference. 17 respondents (73.91 %) felt that the duty hour reform allowed for more successful teaching (3.81 ± 0.35 vs 3.12 ± 0.82, *p* = 0.0009, mean difference 0.67), 16 respondents (69.57 %) felt it allowed for increased proficiency in medical skills (4.04 ± 0.73 vs 3.58 ± 0.88, *p* = 0.001, mean difference 0.46), 15 residents (65.22 %) felt it allowed for more successful learning (4.00 ± 0.56 vs 3.52 ± 0.57, *p* = 0.003, mean difference 0.48), and 14 (60.87 %) felt it caused fewer rotation disruptions (2.84 ± 0.85 vs 3.29 ± 0.88, *p* = 0.04, mean difference −0.45). There was no significant change in perception that resident duty hour reform allowed for more staff supervision, with 7 (30.43 %) perceiving a mean difference of −0.19 (p-value 0.37).

Three out of 5 subdomains in “Senior Resident Wellness” were significant. 13 senior residents (56.52 %) felt that the change in resident duty hours allowed for an improvement in general wellness (3.06 ± 0,71 vs 2.70 ± 0.64, *p* = 0.04, mean difference 0.36), 17 (73.91 %) perceived a decreased exposure to personal harm (2.91 ± 1.06 vs 3.72 ± 0.82, *p* = 0.0003, mean difference −0.8), and 9 (39.13 %) perceived fewer feelings of isolation (2.96 ± 1.07 vs 3.39 ± 1.03, *p* = 0.02, mean difference −0.43). There was no significant change in the perceptions of causing conflicting role demands (mean difference −0.42, *p* = 0.08), or allowing for healthy relationships (mean difference 0.43, *p* = 0.09).

Three out of 5 subdomains in “Perceptions of Ability to Deliver Health Care” revealed significant results. 17 senior residents (73.91 %) believed that the change in duty hours resulted in less potential for error (2.47 ± 0.68 vs 3.15 ± 0.76, *p* = 0.0001, mean difference −0.68), 17 (73.91 %) perceived improvement in clinical skills expertise (3.91 ± 0.52 vs 3.39 ± 0.72, *p* = 0.0004, mean difference 0.52), and 14 residents (60.87 %) perceived increased work efficiency (4.14 ± 0.33 vs 3.72 ± 0.75, *p* = 0.001, mean difference 0.42). There was no significant change in the perception of sub-domains of continuity in patient care (mean difference 0.09, p-value 0.6) and expenditure of emotional labour (mean difference −0.09, p-value 0.58).

## Discussion

In this study, we have used a validated tool to determine changes in resident perceptions after the implementation of a weekend night-float call system. Residents perceived significant improvements in ability to deliver quality health care, resident wellbeing and educational experience as a result of this change.

There has been conflicting evidence regarding the effect of duty hour changes on patient care. Acute and chronic sleep loss has clearly been shown to have deleterious effects on cognitive performance, including psychomotor performance akin to mild alcohol intoxication [18], difficulty with executive functioning, reduced recall and memory, reduced reaction time, and worsening attention and concentration [19]. Therefore, reduced duty hours could result in improved cognitive performance and thus better patient care. A 48-hour work week limit was set in Europe for medical trainees in 2009, and Cappuccio et al. found a 32.7 % decrease in total medical errors, and 82.6 % reduction in intercepted potential adverse events in weekly hours <48 compared to <56 [20]. However, there is conflicting data, because shorter duty hours may increase the number of handoffs between residents, thus possibly introducing additional potential for error. A recent duty hour study of residents in an intensive care unit showed increased adverse events in the group with shorter duty hours, possibly because of the increased number of patient handoffs [14]. In a much larger trial with the assistance of the ACGME, Patel et al. have shown that there was no change in mortality or readmission rates with resident duty hour modification [16]. Indeed in our study, residents did not perceive an improvement in continuity of care as a result of the changes in duty hours.

Given the conflicting research involving duty hour modification, there is a vast need for continued investigation in this area. As per the National Steering Committee of Resident Duty Hours [17], each residency site across Canada should be exploring and researching new and innovative ways to restructure call in an attempt to limit the amount of 24-hour shifts. Our study assesses the outcomes of a novel and distinctive day-float/night-float call model that is unique to Dalhousie University. While we use a validated questionnaire adapted from Fabreau et al. [13] and follow a similar methodology, our call model and residency experience is very different than that of the University of Calgary. Therefore, our study illustrates results from a unique call model that the National Steering Committee of Resident Duty Hours can consider when implementing new models in the future.

Residents did not perceive an improvement in continuity of patient care, which is a major criticism of shortened shift length hours, and a possible source for introduction of error [14, 17]. Potential lack of continuity of care with reduced duty hours may not be a reason to abandon shorter shifts, but a motivation to focus ongoing advancement in handover efficacy and schedule structure, in keeping with the mandate of the National Steering Committee of Resident Duty Hours [13]. Even in this context, our residents perceived improved ability to deliver health care in other domains, including a reduced potential for error, increased clinical skills expertise, and increased work efficiency. This result also correlates with public opinion. Blum et al. in 2010 showed that the American public strongly supports reduction in resident and physician shift length, and would want to be informed if the treating physician were awake >24 h, and furthermore request a different physician at that point [21].

Even if the changes in patient care are uncertain, changes in duty hours on the weekends, may enhance medical learning. A frequent criticism of reduced duty hours involves clinical skills expertise with potentially reduced exposure from shortened shifts, but in our survey residents noted an improvement in their own perception of their clinical skills expertise. Residents felt that a change in duty hours enhanced their medical education experience with more successful teaching and learning, increased proficiency in medical skills, and fewer rotation disruptions. The only sub-domain in medical education experience not affected by the duty hour reform was in regard to staff supervision. This is likely a reflection of the fact that the staff schedule was not changed as part of this intervention.

From an educational perspective, limits on the duration of call shifts have potential benefits beyond the well-known deleterious effects of sleep deprivation and fatigue on cognition. Learning in residency can be considered as a form of *experiential learning*, a process that requires both extensive experience and time to reflect on that experience [22]. Shortened work shifts in a night-float system does not reduce the overall time of learning, but does introduce the opportunity for more “reflection-on-action” (reading, debriefing, self-assessment, etc.) in between shifts, thus potentially enriching the educational experience. Other studies have emphasized that an optimal learning environment likely must contain a balance between work requirements and opportunities for learning and reflection [23].

Resident health and well-being is at least as important to promoting learning as the learning environment itself. Shift length and fatigue have been clearly shown to have detrimental effects upon resident health and well-being, with increased incidence of motor vehicle collisions [1, 3], needle-stick injuries [7], contribution to general fatigue and attention difficulties, and adverse effects on interpersonal relationships [24, 25], as well as physical and mental health [26, 27]. Our study shows that residents perceived a significant improvement in three of 5 sub-domains in resident wellness with implementation of duty hour reform. The two sub-domains that did not change included the resident perception of conflicting role demands and the effects of work hours on their perception of healthy relationships. Although shifts were shorter, the total time that senior residents were in the hospital remained unchanged with the new schedule, and it is possible that total time in hospital is a more important determinant of relationship health and role demands than shift duration.

Resident perception of new call models is important to creating and sustaining new call models. With regard to resident duty hour modification, demonstration of resident perceived benefit is imperative. This is essential for any new call model to become established in a successful post-graduate medical education setting. Our study shows significant perceived improvement, which supports the value of implementing this newly described call model.

## Limitations

A primary limitation of this study is that we record subjective perceptions of the change, rather than objective measurements, such as adverse events in patients or resident performance on validated learning assessments. The study was not designed to address objective evidence of either patient-centered outcomes or skills development. It is a single-center experience of senior internal medicine resident trainees, with a unique local structure of internal medicine emergency department consultation. Other residency training programs and various specialties including surgical and laboratory-based specialties have unique considerations and implications, which could challenge generalization of the results found here. Attending physician and patient opinions were not solicited for the study.

## Conclusions

Resident duty hour reform is occurring at a rapid pace and proper evaluation of new models of call schedules is paramount. As per the recommendations of National Steering Committee on Resident Duty Hours, this study illustrates a successful completion of a pilot duty hour modification. More importantly, it shows that residents had an overall positive perception of the new duty hour reform. There were no sub-domains in which resident perceptions favored the traditional duty hour structure. Overall, residents perceived the greatest improvement in medical education experience. This is an important consideration in a field of research where there are variable results of duty hour reform on patient outcomes. Further study in this area should include objective assessment of clinical performance and learning, patient outcomes, quality of patient handover, and perceptions from other health care professionals.

## Abbreviations

ACGME, Accreditation Council of Graduate Medical Education; RMU, Dalhousie University Research Methods Unit; RPC, Residency Programming Committee; TCPS2, Tri-Council Policy Statement Version 2

## Additional file

Additional file 1: This supplementary material contains the survey that was administered to the senior residents pre- and post-duty hour reform. (DOCX 150 kb)
