# Supplementary material for: Resident duty hour modification affects perceptions in medical education, general wellness, and ability to provide patient care
Source: BMC Med Educ. 2016 Jul 13;16:175. doi: 10.1186/s12909-016-0703-4 (PMC4944256; doi:10.1186/s12909-016-0703-4)
Supplement: Supplementary file 1 — This supplementary material contains the survey that was administered to the senior residents pre- and post-duty hour reform. (DOCX 150 kb) [file 12909_2016_703_MOESM1_ESM.docx]

**Supplementary Material:**

This supplementary material contains the survey that was administered to the senior residents pre- and post-duty hour reform.

| **Impact of Senior Resident Schedule on Senior’s Wellness** | | | | | | |
| --- | --- | --- | --- | --- | --- | --- |
| **Allows General Wellness** |  |  |  |  |  |  |
| **1. Adversely affects my health** |  | **1** | **2** | **3** | **4** | **5** |
| **2. Restricts my participation in physical activity** |  | **1** | **2** | **3** | **4** | **5** |
| **3. Impairs my ability to adapt to Circadian Rhythm changes** |  | **1** | **2** | **3** | **4** | **5** |
| **4. Contributes to overall my fatigue levels** |  | **1** | **2** | **3** | **4** | **5** |
| **5. Contributes to frequent episodes of physical illness (eg. colds)** |  | **1** | **2** | **3** | **4** | **5** |
| **6. Enhances my overall energy levels** |  | **1** | **2** | **3** | **4** | **5** |
| **7. Contributes to my need to use stimulants such as caffeine** |  | **1** | **2** | **3** | **4** | **5** |
| **Allows Exposure to Personal Harm** |  |  |  |  |  |  |
| **8. Impairs safety while driving home post call** |  | **1** | **2** | **3** | **4** | **5** |
| **9. Allows potential for workplace harm (eg. needlestick injuries)** |  | **1** | **2** | **3** | **4** | **5** |
| **Causes Conflicting Role Demands** |  |  |  |  |  |  |
| **10. It is easy for me to trade on-call shifts with others** |  | **1** | **2** | **3** | **4** | **5** |
| **11. Allows me free time to accomplish my non-work related errands** |  | **1** | **2** | **3** | **4** | **5** |
| **12. Provides opportunities to spend time with my family** |  | **1** | **2** | **3** | **4** | **5** |
| **13. Restricts my time available to do research** |  | **1** | **2** | **3** | **4** | **5** |
| **Allows Healthy Relationships** |  |  |  |  |  |  |
| **14. Allows healthy interpersonal relationships** |  | **1** | **2** | **3** | **4** | **5** |
| **Causes feelings of isolation** |  |  |  |  |  |  |
| **15. Causes me to feel isolated at times** |  | **1** | **2** | **3** | **4** | **5** |
| **Impact of Senior Resident Schedule on Ability to Deliver Quality Health Care** | | | | | | |
| **Allows Potential for Error** |  |  |  |  |  |  |
| **16. On the whole, do you feel alert during your procedures while on call** |  | **1** | **2** | **3** | **4** | **5** |
| **17. Do you commit preventable medical errors** |  | **1** | **2** | **3** | **4** | **5** |
| **18. Do you experience “near misses” related to poor patient care** |  | **1** | **2** | **3** | **4** | **5** |
| **19. I’m often too tired to provide safe patient care** |  | **1** | **2** | **3** | **4** | **5** |
| **Allows Clinical Skill Expertise** |  |  |  |  |  |  |
| **20. I miss important diagnoses** |  | **1** | **2** | **3** | **4** | **5** |
| **21. I manage complex medical patients appropriately** |  | **1** | **2** | **3** | **4** | **5** |
| **22. The content of my patient care handover is accurate** |  | **1** | **2** | **3** | **4** | **5** |
| **23. I assume accountability for the patients I admit** |  | **1** | **2** | **3** | **4** | **5** |
| **Causes expenditure of emotional labour** |  |  |  |  |  |  |
| **24. My interactions with other MTU team members are positive** |  | **1** | **2** | **3** | **4** | **5** |
| **25. I communicate well with patients and their families** |  | **1** | **2** | **3** | **4** | **5** |
| **26. I am sensitive to social issues pertaining to patient care (eg. Gender and culture)** |  | **1** | **2** | **3** | **4** | **5** |
| **Allows Work Efficiency** |  |  |  |  |  |  |
| **27. I am able to effectively multitask during busy work times** |  | **1** | **2** | **3** | **4** | **5** |
| **28. I handover patient care issues in a time efficient manner** |  | **1** | **2** | **3** | **4** | **5** |
| **29. I respond to pages in a timely fashion** |  | **1** | **2** | **3** | **4** | **5** |
| **Impact of Senior Resident Schedule on Seniors’ Medical Education Expense** | | | | | | |
| **Allows successful teaching** |  |  |  |  |  |  |
| **30. I have enough time to teach junior residents and clerks** |  | **1** | **2** | **3** | **4** | **5** |
| **31. I have enough energy to teach junior residents and clerks** |  | **1** | **2** | **3** | **4** | **5** |
| **32. I am confident in my ability to teach procedural skills** |  | **1** | **2** | **3** | **4** | **5** |
| **33. I am confident in my ability to teach how to manage unstable critically ill patients** |  | **1** | **2** | **3** | **4** | **5** |
| **34. I am confident in my ability to teach the skills of how to run a code** |  | **1** | **2** | **3** | **4** | **5** |
| **Allows Medical Skills Proficiency** |  |  |  |  |  |  |
| **35. I am confident in my ability to perform procedures** |  | **1** | **2** | **3** | **4** | **5** |
| **36. I am confident in my ability to manage unstable critically ill patients** |  | **1** | **2** | **3** | **4** | **5** |
| **37. I am confident in my ability to run a code** |  | **1** | **2** | **3** | **4** | **5** |
| **Allows Successful Learning** |  |  |  |  |  |  |
| **38. I can acquire new knowledge on call** |  | **1** | **2** | **3** | **4** | **5** |
| **39. I can retain new knowledge on call and apply it to patient care** |  | **1** | **2** | **3** | **4** | **5** |
| **40. My overall education experience on call is satisfying** |  | **1** | **2** | **3** | **4** | **5** |
| **41. I have opportunities to learn procedures through simulation training** |  | **1** | **2** | **3** | **4** | **5** |
| **Allows Staff Physician Supervision** |  |  |  |  |  |  |
| **42. I have the opportunity to review cases with attending physicians** |  | **1** | **2** | **3** | **4** | **5** |
| **43. My clinical skills (history and physical) are observed by an attending physician** |  | **1** | **2** | **3** | **4** | **5** |
| **44. I received feedback from attending physicians** |  | **1** | **2** | **3** | **4** | **5** |
| **Causes rotation disruptions** |  |  |  |  |  |  |
| **45. My ambulatory care rotations are frequently interrupted due to MTU on call duties** |  | **1** | **2** | **3** | **4** | **5** |
| **46. I am post-call more often** |  | **1** | **2** | **3** | **4** | **5** |
| **47. I am tired after a weekend of call and it affects my week-day rotations** |  | **1** | **2** | **3** | **4** | **5** |
